# A full-thickness spatial transcriptomics atlas of medically refractory ulcerative colitis identifies niche signatures associated with biologic non-response

**DOI:** 10.3389/fimmu.2026.1929046

**Published:** 2026-09-25

**Authors:** Qingquan Bai, Runyang Li, Zhengjin Liu, Yuying Zhang, Xiao He, Junjie Yao, Shengdong Wu, Jing Huang, Shuqi Mao, Yunzheng Zhao, Yijia Chen, Linwei Wang, Yueping Zheng, Yanyan Zhan, Tianhui Hu, Michael Sigal, Frank Tacke, Caide Lu, Cornelius Engelmann, Yiqun Hu

**Affiliations:** 1 Department of Hepatobiliary and Pancreatic Surgery, Ningbo Medical Centre Lihuili Hospital, Affiliated Lihuili Hospital of Ningbo University, Ningbo, China; 2 Department of Hepatology and Gastroenterology, Charité – Universitätsmedizin Berlin, Campus Virchow-Klinikum and Campus Charité Mitte, Berlin, Germany; 3 Cancer Research Centre, School of Medicine, Xiamen University, Xiamen, China; 4 Department of Pathology, Zhongshan Hospital of Xiamen University, School of Medicine, Xiamen University, Xiamen, China; 5 School of Life Sciences, Xiamen University, Xiamen, China; 6 Department of Neurology, Ningbo Medical Centre Lihuili Hospital, Ningbo University, Ningbo, China; 7 School of Basic Medical Sciences, Fujian Medical University, Fuzhou, China; 8 Cancer Department, The Second Hospital of Longyan, Longyan, China; 9 Department of Gastroenterology, Zhongshan Hospital of Xiamen University, School of Medicine, Xiamen University, Xiamen, China

**Keywords:** biologic non-response, cellular niche, spatial transcriptomics, tertiary lymphoid structures, ulcerative colitis

## Abstract

**Background:**

Ulcerative colitis (UC) is a chronic mucosal immune disorder in which 30-50% of patients fail biologic therapy, yet the full-thickness immune architecture of medically refractory disease and reliable tissue-level markers of treatment response remain undefined.

**Methods:**

Using 10x Visium HD spatial transcriptomics (16-µm bins; 615,274 bins), we generated a full-thickness spatial atlas of surgically resected colon from patients with medically refractory UC (n=3) and controls (n=3), integrating niche-stratified differential expression, spatial cell–cell communication, SCENIC regulon inference and single-sample GSEA with immunofluorescence and DSS-colitis flow cytometry.

**Results:**

We resolved 18 spatial immune–stromal niches across five tissue modules. At the mucosal frontline, epithelial barrier collapse was accompanied by a 20-fold expansion of ulcer-bed stroma (p=0.030) that co-localised with an Acute Neutrophil Niche and was associated with neutrophil recruitment via CXCL12–CXCR4 signalling, forming a spatially integrated ulcerogenic unit. In the submucosa, a humoral immune programme was associated with tertiary lymphoid structures (TLS) and plasma-cell survival-related signalling (MIF–CD74 and LTB–CD40). This niche architecture was immunologically informative: an Acute Neutrophil Niche signature was enriched in biologic non-responders across six adult cohorts (AUC 0.67–0.99) and in non-remission patients of the paediatric PROTECT cohort, and a five-niche model showed discriminative performance in adult cohorts across independent datasets and drug classes (anti-TNF and anti-integrin; cross-cohort AUC 0.68–0.97), although this performance did not transfer to the paediatric cohort (AUC ≈ 0.51–0.62). Expansion of TNFAIP2+ TLS-resident B cells was confirmed at the protein level in a DSS colitis mouse model by immunofluorescence and flow cytometry (p=0.0079).

**Conclusion:**

Together, this full-thickness atlas defines the spatial immune ecosystem of refractory UC – neutrophil niches, TLS and plasma-cell survival programmes – and yields cellular-niche signatures reproducibly associated with treatment outcome across drug classes, providing an immunologically grounded framework that may inform biologic selection, pending prospective validation.

## 1 Introduction

Ulcerative colitis (UC) is a chronic, relapsing inflammatory bowel disease (IBD) characterised by diffuse mucosal inflammation of the colon and rectum, affecting millions of individuals worldwide with rapidly rising incidence in newly industrialising regions (1). The disease imposes a substantial burden on patients and healthcare systems, with up to 15% of affected individuals ultimately requiring total colectomy for medically refractory disease or neoplastic complications (2). Although the pathogenesis of UC involves a multifactorial interplay between genetic predisposition, environmental triggers, dysregulated immune responses, and epithelial barrier dysfunction, the precise cellular and molecular mechanisms that drive the irreversible architectural collapse characteristic of refractory disease remain incompletely understood (2).

The advent of biologic therapies — including anti-TNF agents (infliximab, adalimumab) (3), anti-integrin antibodies (vedolizumab) (4), Janus kinase (JAK) inhibitors (tofacitinib, upadacitinib), and IL-12/23 inhibitors (5) — has transformed the treatment landscape of moderate-to-severe UC. Nevertheless, primary non-response and secondary loss of response remain major clinical challenges, with approximately 30–50% of patients failing to achieve sustained remission with any given biologic agent (2). The lack of reliable molecular biomarkers to predict treatment response at the tissue level has been a critical barrier to personalised therapeutic decision-making in UC.

Single-cell RNA sequencing (scRNA-seq) has provided high-resolution insight into the transcriptomic heterogeneity of the UC colon. Landmark studies have mapped the cellular diversity of the colonic mucosa in health and disease, revealing disease-associated epithelial and stromal cell states, and identifying inflammatory fibroblast subpopulations associated with anti-TNF non-response (6). However, conventional scRNA-seq inherently sacrifices spatial context during tissue dissociation, precluding direct interrogation of the spatially organised cellular ecosystems — from the mucosal epithelial surface to the deep muscularis propria — that define UC pathology across all tissue layers.

Spatial transcriptomics has emerged as a transformative technology that enables transcriptome-wide gene expression profiling whilst preserving two-dimensional spatial coordinates within intact tissue sections (7). The 10x Visium HD platform advances this capability to sub-cellular bin resolution, enabling spatially resolved interrogation of tissue architecture at high resolution. Whilst initial spatial transcriptomics studies in IBD have begun to map immune cell heterogeneity and cell-communication networks in mucosal biopsies (8), these analyses have predominantly focused on endoscopic biopsy specimens with limited tissue depth, leaving the full-thickness spatial organisation of refractory surgical UC — including the critical submucosal and mural compartments — largely unexplored.

Here, we show, through comprehensive high-definition spatial transcriptomics of full-thickness colonic resection specimens from three medically refractory UC patients (total colectomy, Mayo score ≥ 10) and three normal controls, that the refractory UC colon is organised into 18 spatially distinct functional niches across five hierarchical tissue modules. By integrating zone-stratified transcriptomic analyses, spatial cell-cell communication mapping, and SCENIC transcription factor regulon inference (9) using Squidpy (10), we reveal spatially integrated pathological programmes. Spatial niche gene expression signatures derived from this atlas associate with biologic non-response and clinical remission across seven independent clinical cohorts spanning multiple drug classes. Together, our findings provide a high-resolution spatial map of the pathological microenvironment in refractory UC and identify spatially grounded transcriptional signatures that may inform biologic therapy selection.

The central discovery of this atlas is a single pathological unit at the mucosal frontline: an ulcer-bed-centred tri-niche axis in which ulcer bed stroma (C1), acute neutrophils (C14) and hyperemic vasculature (C5) co-localise and expand at the expense of the epithelial barrier, giving rise to niche signatures that associate with biologic non-response across independent cohorts. The submucosal humoral programme (tertiary lymphoid structures and plasma cells) and the regulon architecture are presented as integral components of this atlas, contextualising this core unit within the full-thickness disease architecture.

## 2 Materials and methods

### 2.1 Human subjects and tissue collection

Human colon tissues were obtained from Zhongshan Hospital of Xiamen University, including 3 non-IBD surgical controls, hereafter referred to as normal controls — full-thickness normal colon resected for non-IBD indications and confirmed histologically normal, with no history of IBD or other chronic inflammatory bowel disease (Mayo score 0) — and 3 severe, treatment-refractory UC tissues (Mayo score ≥ 10). Here and throughout the manuscript, the term “normal” refers to the tissue phenotype (histologically non-inflamed colon without evidence of colitis), not to the systemic health status of the donors, consistent with the standard convention in surgical tissue studies. All specimens were collected with written informed consent (Ethics Approval No. 2025-066). Samples were fixed in 4% paraformaldehyde, dehydrated, cleared and paraffin-embedded. Serial sections from the same blocks were used for both 10x Visium HD spatial transcriptomics and histopathological assessment. Detailed sample-level clinical and demographic characteristics for all six specimens (gender, age, height, weight, body mass index [BMI], Mayo score and endoscopic Mayo subscore) are provided in **Supplementary Table S1**.

### 2.2 10x Visium HD library preparation and sequencing

Sections (5 µm) were processed following the 10x Genomics Visium HD FFPE protocol, using the Visium HD Human Transcriptome Probe Set v2.0 (probe-based chemistry) and the Visium CytAssist instrument. Libraries were sequenced on an Illumina NovaSeq 6000 platform. The 16 μm square bin was used for all downstream analyses.

### 2.3 Spatial transcriptomics data preprocessing and quality control

Raw reads were aligned to GRCh38 using Space Ranger. Quality control removed bins with <50 counts or >25% mitochondrial gene fraction, retaining 615,274 of the 850,448 raw bins (≈72%) for downstream analysis; per-sample raw bin counts are listed in **Supplementary Table S1**. The top 3,000 highly variable genes were selected using the Seurat v3 dispersion method (11).

### 2.4 Cell-type deconvolution by Tangram

Spatial cell-type proportions were inferred using Tangram (v1.0.3) (12), which maps single-cell RNA-seq reference profiles onto Visium HD spatial spots by minimising the spatial correlation loss between the mapped and measured transcriptomes. The single-cell reference was constructed by integrating five publicly available human colon scRNA-seq datasets: the UC colon atlas (34,772 cells; CELLxGENE) (6); the Pan-GI Tract Atlas (96,675 colon-derived cells from 25 integrated gut atlases) (13); the UC-specific subset of the Pan-GI Atlas (23,904 cells) (13); a UC CITE-seq dataset (GSE250490; ~20,000 cells) (14); and the Gut Cell Atlas colon reference (~100,000 cells) (15). Datasets were downloaded from CELLxGENE and GEO, and all gene identifiers were unified to HGNC symbols. After per-dataset quality control (cells with <200 or >6,000 detected genes, or >20% mitochondrial reads, were removed), the five datasets were concatenated and batch-corrected using Harmony (16). The integrated reference was annotated at two levels: 6 broad cell types (Epithelial, Stromal, Myeloid, Lymphoid, B_Plasma, Neural) and 11 fine cell types (B_Plasma, CD4_T, CD8_T, Epithelial, ILC, Myeloid, NK, Neural, Stromal, Th17, Treg), yielding a balanced reference of 11,000 cells (1,000 per fine cell type). The top 3,000 highly variable genes were selected to match the spatial data. Tangram mapping was performed on the complete Visium HD dataset (615,274 spots × 3,000 HVGs) with the 11-cell-type reference using default parameters. Because Tangram deconvolution accuracy is reduced in the muscularis propria and serosa layers where the reference single-cell data poorly represent smooth-muscle and mesothelial cell states, all cell-type-level downstream analyses were restricted to the mucosa and submucosa zones (363,939 spots; 59.2% of total), which contain UC-relevant pathology and show high spatial coherence between deconvolved cell-type proportions and histological zone annotations.

### 2.5 Multi-sample integration, batch correction, and clustering

Expression matrices from all six samples were concatenated (615,274 bins). PCA (n_components = 50) was performed, and batch effects were corrected using Harmony (16). Leiden clustering (resolution = 1.2) resolved 18 stable niches.

### 2.6 Niche annotation and spatial zone assignment

Each cluster was annotated by manual inspection of marker genes cross-referenced against the Human Cell Atlas (15). Histological zones were assigned via pathologist-annotated H&E registration using QuPath v0.5.

### 2.7 Zone-stratified differential expression analysis

Differential expression between UC and normal colon was performed using two-sided Wilcoxon rank-sum test on spatially defined bins (all UC bins versus all normal bins within each histological zone). Genes with |logFC| ≥ 0.5 and adjusted p < 0.05 were considered significant, consistent with the threshold used in zone-stratified analyses. We acknowledge that, with three biological replicates per group, the statistical power of this comparison is limited, and that spatial bins within a section are not independent observations; all differentially expressed genes identified in this discovery cohort are therefore interpreted as descriptive and hypothesis-generating rather than definitive.

### 2.8 Gene set enrichment analysis

GSEA was performed using GSEApy with Reactome and GO-BP databases. Pathways with FDR < 0.05 were considered significant.

### 2.9 Spatial ligand-receptor and neighbourhood enrichment analysis

Ligand-receptor analysis was performed using Squidpy (10) with the OmniPath database; only interactions with permutation-based p < 0.05 (1,000 permutations) were considered. Neighbourhood enrichment was computed using sq.gr.nhood_enrichment.

### 2.10 SCENIC transcription factor regulon analysis

Regulon inference was performed using pySCENIC v0.12.1 (9) with GRNBoost2 and cisTarget hg38 v10 databases. AUCell scores were Z-score normalised across niches.

### 2.11 External cohort ssGSEA analyses

Per-niche signatures (up to 30 marker genes per niche, derived from the niche-level differential expression analysis; complete gene lists in **Supplementary Table S3**) were evaluated across six GEO series comprising seven independent clinical cohorts. ssGSEA was computed using GSEApy. Comparisons used two-sided Mann-Whitney U tests with Benjamini-Hochberg FDR correction.

### 2.12 ROC analysis and multivariable modelling

For each niche signature and cohort, ssGSEA scores were used as a continuous variable in ROC analysis of biologic non-response (or non-remission for GSE109142). ROC curves and areas under the curve (AUC) were computed, with 95% confidence intervals estimated by 1,000-fold bootstrap resampling and optimal cut-offs defined by the Youden index. A multivariable logistic model combining the C14, C1, C5, C6 and C15 signatures was fitted and evaluated by leave-one-out cross-validation within each cohort. Transferability across cohorts was assessed by training the model in one cohort and applying it, without refitting, to each remaining cohort (cross-cohort transfer). Analyses were performed with scikit-learn; p values were corrected using the Benjamini–Hochberg procedure.

### 2.13 DSS-induced colitis mouse model

Male C57BL/6J mice were randomly assigned to control (n = 15) or 3% DSS (n = 20) for 7 days, as previously described (17). For flow cytometric analysis, a randomly selected subset of mice (n = 5 per group) was euthanised on day 10 by CO_2_ inhalation (30% chamber volume per minute displacement rate), followed by cervical dislocation as a secondary confirmatory method and used for downstream profiling; the remaining animals were used for parallel histological analyses. All procedures were approved by the Animal Ethics Committee of Xiamen University (XMULAC20220195).

### 2.14 Histopathological processing and immunofluorescence

Tissue samples were fixed, dehydrated, cleared, and embedded in paraffin. Sections were cut at 4 μm thickness. For immunofluorescence (IF), antigen retrieval was performed in EDTA buffer (pH 9.0) under heated conditions on mouse colon sections, which were blocked and incubated overnight at 4 °C with the following primary antibodies: CD19 (Thermo Scientific, Cat# 14-0199-82, 1:200) and TNFAIP2 (Abcepta, Cat# AP6593c, 1:100). After washing, sections were incubated with species-appropriate secondary antibodies: Cy3-conjugated goat anti-mouse IgG (Invitrogen, Cat# A10521, 1:400) and FITC-conjugated goat anti-rabbit IgG (Invitrogen, Cat# F2765, 1:400). Nuclei were counterstained with DAPI. Images were acquired using a Nikon Fi3 fluorescence microscope, and scale bars (20 µm) are shown in all immunofluorescence panels.

### 2.15 Flow cytometric analysis

Colon tissues were mechanically dissociated and digested in 0.2% collagenase solution. Single-cell suspensions were filtered through a 70-μm strainer and stained as follows. For surface antigen detection, cells were incubated with anti-CD19 antibody (Thermo Scientific, Cat# 14-0199-82, 1:200) followed by Cy3-conjugated goat anti-mouse IgG (SouthernBiotech, Cat# 0100-01). For intracellular staining, cells were fixed, permeabilised, and incubated with anti-TNFAIP2 antibody (Abcepta, Cat# AP6593c, 1:100) followed by FITC-conjugated goat anti-rabbit IgG (Thermo Fisher Scientific, Cat# F2765). Data acquisition was performed on a CytoFLEX flow cytometer (Beckman Coulter), and data were analysed using CytExpert software (Beckman Coulter).

### 2.16 Software environment and statistical analysis

All analyses were performed in Python 3.11 using Scanpy (18) v1.10, Squidpy (10) v1.4, GSEApy, pySCENIC (9) v0.12.1, and associated libraries. Nominal p < 0.05 was considered statistically significant with Benjamini-Hochberg correction for multiple testing.

## 3 Results

### 3.1 Spatial transcriptomics resolves 18 niches in refractory UC

To construct a comprehensive, spatially resolved atlas of the colonic microenvironment in refractory ulcerative colitis (UC), we performed 10x Visium HD spatial transcriptomics on full-thickness colonic resection specimens from three patients with medically refractory UC requiring total colectomy and three normal controls (n = 3 per group; **Supplementary Table S1**). Unsupervised graph-based clustering (Leiden algorithm, resolution 1.2) of all spatially barcoded spots, following integrated batch correction across samples, resolved the colonic tissue into 18 transcriptionally distinct niches (Clusters 0–17; **Figure 1A**). Manual annotation based on canonical marker gene signatures assigned cell-type identities: Ulcer Bed Stroma (C1; IGHG1, COL1A1, MMP2), Acute Neutrophil Niche (C14; S100A8, S100A9, CXCL8), Lymphoid Follicles/TLS (C16; CCL19, CXCL13, MS4A1), Mature Enterocytes (C6; CEACAM5, SLC26A3, FABP1), Plasma Cells (C13; IGKC, IGHA1, JCHAIN), Goblet Cells (C15; MUC12, PIGR, FCGBP), and Activated Macrophages (C17; CD74, LYZ, C1QC), amongst others (**Figure 1B**). UMAP visualisation coloured by disease condition revealed a pronounced global transcriptomic divergence between UC and normal colon, with UC-specific spots selectively enriched within the pathological niches (**Figure 1C**). This global separation reflects the dominant disease-associated transcriptomic remodelling documented in Section 3.2 (8,380 genes downregulated in the mucosa) and is therefore expected rather than contradictory to the identification of shared niches: clustering was performed on the Harmony-corrected high-dimensional PCA embedding, whereas the UMAP in **Figures 1A, C** is a two-dimensional visualisation of the same embedding. Consistent with shared functional units, all 18 niches were detected in both UC and normal colon, albeit with markedly different abundances (**Supplementary Table S2**), with pathological niches expanded in UC (e.g., 16,123 vs. 987 ulcer-bed-stroma spots and 1,242 vs. 26 acute-neutrophil spots in the UC vs. normal mucosa) and epithelial niches enriched in normal colon. These 18 niches were hierarchically organised into five overarching functional modules — Pathological Core (C1, C14, C16), Immunity & Barrier (C6, C13, C15, C17), Stroma & Vasculature (C3, C5, C7, C9, C11), Muscle Layer (C0, C2, C4, C8), and Nervous & Adipose Tissue (C10, C12) — that organised the transcriptomic landscape into coherent biological units. Spatial zone inference accurately recapitulated the classic histological stratification of the colon — Mucosa, Submucosa, Muscularis Propria, and Serosa — as validated against H&E-stained serial sections (**Figure 1D**; **Supplementary Figure S1A**), with the five functional modules concordantly mapped onto their native histological context (**Figure 1E**). Spatial feature mapping at single-niche resolution further confirmed that the Pathological Core niches were strongly expanded at the denuded mucosal frontline and in the deep submucosa of UC specimens (**Figure 1F**), consistent with the spatial specificity of the identified pathological niches. Orthogonal validation by Tangram deconvolution, which maps single-cell reference profiles onto each spatial spot, confirmed these niche assignments: deconvolved Epithelial proportions were selectively enriched in Mature Enterocyte (C6) and Goblet Cell (C15) niches, Myeloid proportions in the Acute Neutrophil Niche (C14), and B_Plasma proportions in Plasma Cell (C13) and TLS (C16) niches (**Supplementary Figure S1B**), independently corroborating the transcriptional niche identities.

**Figure 1:**
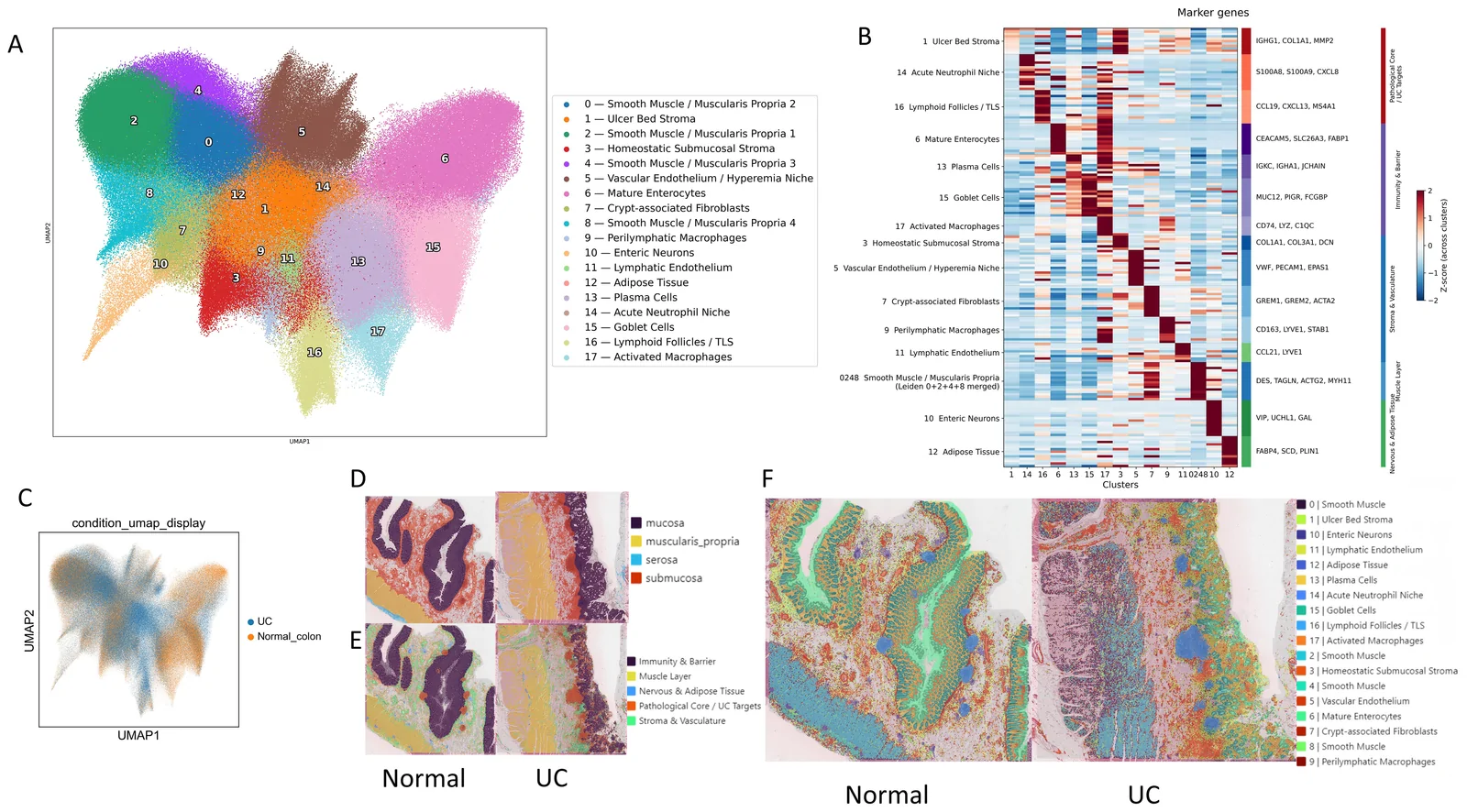
High-definition spatial transcriptomics delineates 18 functional niches in the colonic microenvironment of refractory ulcerative colitis. **(A)** UMAP plot visualising the 18 transcriptionally distinct niches. **(B)** Heatmap of top cluster-specific marker genes. **(C)** UMAP embedding of all spatial spots coloured by disease condition (UC, n = 3; Normal colon, n = 3). **(D)** Spatial maps of histological zones on H&E-stained tissue sections. **(E)** Spatial maps of five functional modules on H&E-stained tissue sections. **(F)** Spatial maps of 18 fine niche annotations on H&E-stained tissue sections.

### 3.2 Mucosal barrier collapse and ulcer bed formation in UC

Zone-stratified differential expression analysis of the spatially defined mucosa revealed extensive transcriptional remodelling in refractory UC: 8,380 genes were significantly downregulated compared to only 213 upregulated genes (|logFC| ≥ 0.5, FDR < 0.05; **Figure 2A**). The most profoundly suppressed transcripts included canonical absorptive colonocyte identity markers (AQP8, GUCA2A, GUCA2B, SLC26A2, CA1) and mitochondrially encoded transcripts (MT1G, MT-ND3), consistent with a profound loss of epithelial cell identity and a shift in metabolic programmes. In sharp contrast, the upregulated gene set was dominated by the tissue-destructive matrix metalloproteinases MMP1, MMP3, and MMP7, the neutrophil chemoattractants CXCL5 and CSF3, and the innate immune antimicrobial peptides REG1A and REG3A (**Figure 2A**). Gene Set Enrichment Analysis (GSEA, Reactome) confirmed robust enrichment of extracellular matrix (ECM) degradation and MMP activation pathways amongst upregulated gene sets, whilst negatively enriched pathways documented profound breakdown of tight junction and adherens junction complexes alongside a collapse of aerobic metabolic programmes (**Figure 2C**). These transcriptional and spatial findings were further quantified by spatial proportion analysis (**Figures 2E, F**): the two principal epithelial barrier-forming populations — Mature Enterocytes (C6) and Goblet Cells (C15) — were markedly depleted in the UC mucosa compared to normal colon (C6: 14.89% vs. 25.44%, Δ = −10.55%, p = 0.004; Goblet Cells (C15): 16.59% vs. 30.55%, Δ = −13.96%, p = 0.004; two-sided t-test), whilst three pathological niches underwent significant expansion: the Ulcer Bed Stroma (C1) increased from 0.91% in normal colon to 18.36% in UC (Δ = +17.44%, p = 0.030), representing a 20-fold absolute expansion of the ulcerogenic stroma at the cost of the epithelial monolayer; the Vascular Endothelium/Hyperemia Niche (C5) also expanded significantly (4.20% vs. 1.05%, Δ = +3.16%, p = 0.004), consistent with the pathological neovascularisation and hyperemia characteristic of active UC ulceration. Of note, Activated Macrophages (C17) were paradoxically depleted in the UC mucosa (1.82% vs. 5.76%, Δ = −3.94%, p = 0.006), likely reflecting their redistribution to deeper tissue compartments.

**Figure 2:**
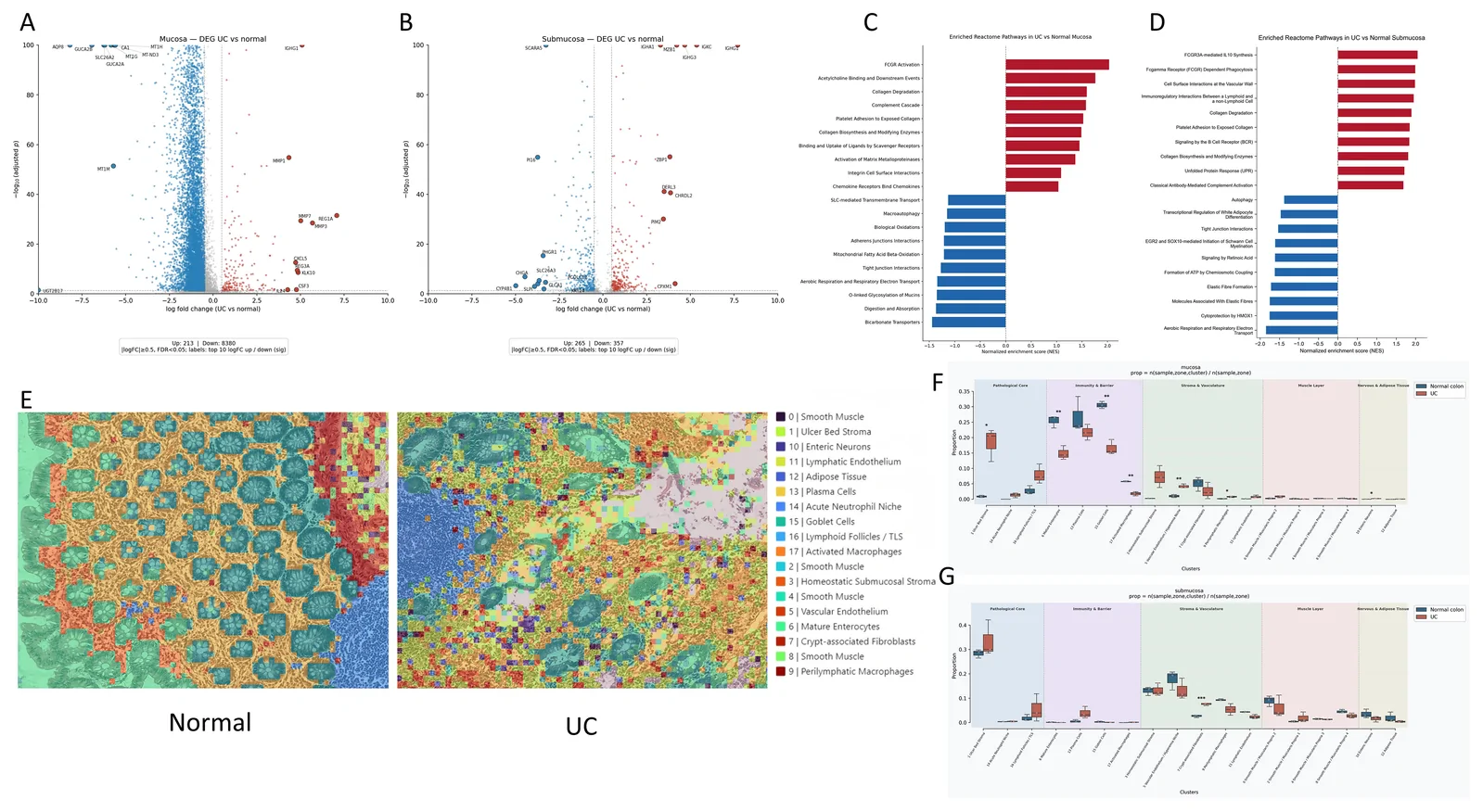
Spatial zone-stratified transcriptomics reveals compartment-specific remodelling in refractory UC: catastrophic mucosal barrier collapse and submucosal humoral immune storm. **(A)** Volcano plot of mucosal DEGs (UC vs. Normal; Up: 213, Down: 8,380). **(B)** Volcano plot of submucosal DEGs (Up: 265, Down: 357). **(C)** GSEA (Reactome) of mucosal DEGs. **(D)** GSEA (Reactome) of submucosal DEGs. **(E)** Spatial zoom-in maps of mucosal regions in normal colon and UC. **(F)** Boxplots of cluster spatial proportions within the mucosa zone (UC vs. Normal, n = 3 per group). **(G)** Boxplots of cluster spatial proportions within the submucosa zone. *p < 0.05; **p < 0.01; ***p < 0.001 (two-sided t-test).

### 3.3 Submucosal humoral immune activation in UC

In contrast to the mucosal transcriptional collapse, the submucosa of refractory UC specimens exhibited a more balanced, bidirectional DEG landscape (265 upregulated vs. 357 downregulated genes; **Figure 2B**), indicative of active immune reorganisation rather than purely destructive remodelling. The upregulated gene set was dominated by a profound humoral immune activation: the top upregulated transcripts comprised immunoglobulin-encoding genes (IGHA1, IGKC, IGHG1, IGHG3) and the endoplasmic reticulum (ER)-resident chaperone MZB1, collectively indicative of strong plasma cell infiltration and high-volume antibody secretion. Additional upregulated transcripts included the innate immune sensor ZBP1, the ER-associated degradation component DERL3, and the plasma cell pro-survival kinase PIM2, reflecting concurrent activation of innate inflammatory sensing and the unfolded protein response (UPR) imposed by the extraordinary secretory burden. Conversely, the homeostatic submucosal stromal markers SCARA5 and PI16 were profoundly downregulated, indicating dissolution of the normal submucosal niche architecture (**Figure 2B**). GSEA of submucosal DEGs confirmed activation of B cell receptor signalling and the UPR as the most significantly enriched upregulated pathways, corroborating high-volume immunoglobulin synthesis by terminally differentiated plasma cells. FcγR-dependent phagocytosis was also enriched, indicating active clearance of inflammatory debris by submucosal macrophages (**Figure 2D**). Quantitative spatial proportion analysis within the submucosa zone further revealed significant expansion of Crypt-associated Fibroblasts (**Figure 2G**).

### 3.4 Communication collapse and signalling rewiring in UC

To systematically characterise the intercellular signalling rewiring that accompanies refractory UC, we performed spatial ligand-receptor interaction analysis across all 18 identified niches. Global communication network analysis revealed a profound “communication desertification” in refractory UC: the total cumulative intercellular interaction weight was markedly reduced compared to the highly interconnected, homogeneous network of normal colon (**Figure 3A**), reflecting the systemic collapse of paracrine signalling as tissue architecture disintegrated. The few remaining high-weight interactions in UC were concentrated within the pathological niches, consistent with a contraction of signalling towards pathological programmes. Spatial neighbourhood enrichment analysis (Z-score heatmap, 8 key niches; **Figure 3B**) supported the pathological compartmentalisation of refractory UC through two mutually reinforcing spatial co-localisation axes. A pathological inflammatory axis was identified, characterised by strong positive spatial co-enrichment between Ulcer Bed Stroma (C1) and Acute Neutrophil Niche (C14), forming a spatially co-localised ulcerogenic unit at the mucosal frontline. Although Lymphoid Follicles/TLS (C16) and Plasma Cells (C13) were both anatomically restricted to the submucosa (**Figure 1F**, **2E**), neighbourhood enrichment indicated that TLS formed discrete, self-contained aggregates (the highest self-enrichment Z-score amongst all niches), whereas Plasma Cells preferentially co-localised with Activated Macrophages (C13–C17 Z = +101.5), suggesting that the TLS–Plasma Cell survival programme operates predominantly through paracrine rather than direct contact-mediated signalling. We confirmed that the ECM-dominated communication landscape is not an artefact of highly variable gene (HVG) selection: full-gene LIANA analysis using all 18,085 detected genes (recovering 1,748 consensus ligand-receptor genes vs. 577 in the 3,000-HVG set) showed that canonical inflammatory cytokine mRNAs (IL1B, TNF, IL6) are expressed in fewer than 1% of Visium HD spots, whereas collagen genes (COL1A1, COL3A1) are detected in 30–53% of spots, indicating that the ECM-dominated myeloid communication landscape reflects the transcript-detection characteristics of this platform rather than a bias introduced by HVG selection. Analysis of specific ligand-receptor pairs revealed coherent rewiring of intercellular communication at both the mucosal frontline and the deep immune niche (**Figures 3C–F**). The homeostatic stromal-epithelial maintenance axis between Homeostatic Submucosal Stroma (C3) and Mature Enterocytes (C6) — normally supported by interactions including DCN–EGFR — was selectively attenuated in refractory UC, reflecting the loss of epithelial trophic support (**Figure 3C**). In its place, the Ulcer Bed Stroma (C1) showed a signalling output shifted towards pathological effectors: CXCL12–CXCR4 and COL6A3–CD44 interactions were associated with neutrophil recruitment and retention within the ulcer bed microenvironment (C1→C14; **Figure 3D**), whilst VEGFA–CDH5 signalling was associated with vascular hyperemia and endothelial permeability at the ulcer–vessel interface (C1→C5; **Figure 3E**). Despite the global communication collapse, the TLS→Plasma Cell axis (C16→C13) selectively retained survival-promoting signals — MIF–CD74 (anti-apoptotic) and LTB–CD40 (B cell differentiation and activation) — consistent with plasma cell persistence and high-volume antibody production within the fragmented submucosal environment (**Figure 3F**).

**Figure 3:**
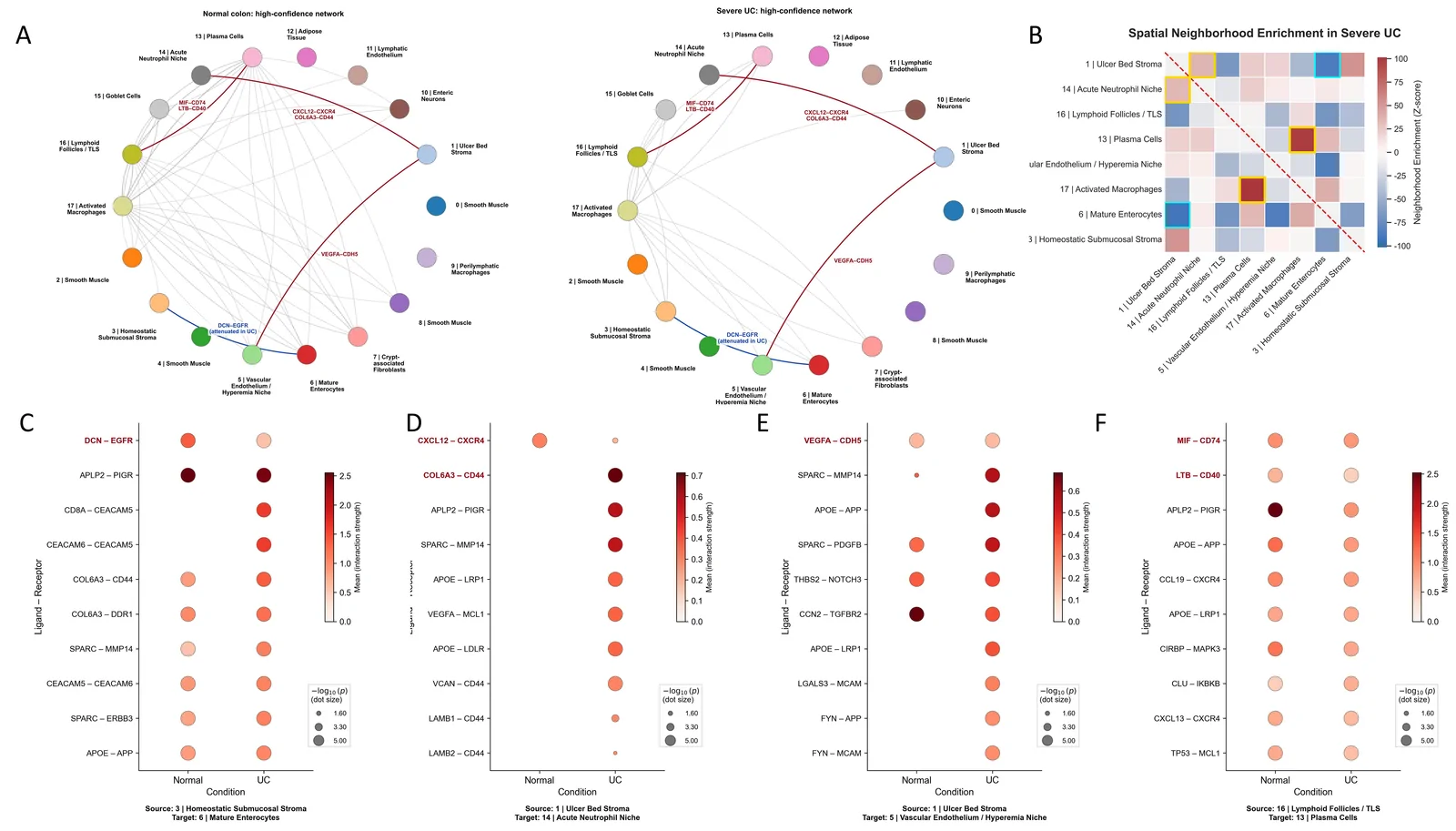
Spatial cell-cell communication analysis in refractory UC. **(A)** High-confidence ligand-receptor (L-R) interactions of the 18-niche network, shown separately for normal colon and severe UC on a shared scale. Only interactions passing permutation testing (p < 0.05, 1,000 permutations) with >= 900 significant L-R pairs are shown; edge width and opacity are proportional to interaction weight on a common scale. Red: pathological signalling axes enhanced in UC (CXCL12-CXCR4/COL6A3-CD44; VEGFA-CDH5; MIF-CD74, LTB-CD40); blue: homeostatic axis attenuated in UC (DCN-EGFR). **(B)** Spatial neighbourhood enrichment heatmap (Z-score, 8 key niches); yellow frames highlight the co-enrichment of the ulcer-bed/neutrophil, ulcer-bed/vascular, and TLS/plasma-cell axes. **(C–F)** Top 10 L-R pairs per axis: **(C)** Homeostatic Submucosal Stroma to Mature Enterocytes, **(D)** Ulcer Bed Stroma to Acute Neutrophil Niche, **(E)** Ulcer Bed Stroma to Vascular Endothelium/Hyperemia Niche, **(F)** Lymphoid Follicles/TLS to Plasma Cells. Pairs highlighted in bold red are the signalling axes discussed in the text; dot colour = mean interaction strength, dot size = -log10(p).

### 3.5 Master transcription factors of pathological niches

To define the upstream gene regulatory networks underlying the identified pathological niches, we applied the SCENIC framework (Single-Cell rEgulatory Network Inference and Clustering) to infer transcription factor (TF) regulon activities from all spatial transcriptomic spots. A comprehensive regulon atlas encompassing the top-5 TFs per niche (90 TFs total) revealed that each of the 18 spatially defined niches is governed by a highly specific and largely non-overlapping master regulator programme (**Figure 4A**). Hierarchical clustering of regulon activities faithfully recapitulated the predefined functional module structure: Smooth Muscle clusters (C0, C2, C4, C8) shared a conserved myogenic regulatory axis, whilst Enteric Neurons (C10) displayed the most transcriptionally distinct regulon fingerprint of all 18 niches, underscoring the analytical sensitivity and biological resolution of the spatial regulon atlas. Restricting analysis to the five key disease-associated niches (Ulcer Bed Stroma C1, Acute Neutrophil Niche C14, Plasma Cells C13, Lymphoid Follicles/TLS C16, and Activated Macrophages C17) revealed largely distinct transcriptional regulatory programmes (**Figure 4B**). The Ulcer Bed Stroma (C1) was uniquely characterised by hyperactivation of the ELK3 (angiogenesis and ETS-family stress response) and NFE2L2 (oxidative stress response) regulons, alongside MAFB and HDX, collectively representing an adaptive pro-survival and tissue-destructive programme operative under the extreme oxidative and hypoxic microenvironment of the ulcer bed. The Acute Neutrophil Niche (C14) was governed predominantly by SPI1, the canonical master regulator of myeloid lineage identity and neutrophil effector function, together with FOXO1 and EBF4 as additional top-ranked regulons supporting neutrophil survival and stress adaptation within the inflammatory milieu. Plasma Cells (C13) were associated with elevated FOXC2, PTF1A, and ALX3 regulon activity, regulons that collectively support the extraordinary antibody-secretory capacity of terminally differentiated plasma cells. Lymphoid Follicles/TLS (C16) exhibited activation of NR3C1, RORA, NFAT5, HES7, and FOXC2, regulons classically associated with glucocorticoid-mediated lymphocyte regulation, germinal centre programming, osmoregulated lymphocyte activation, and lymphoid organogenesis. Activated Macrophages (C17) displayed a distinct regulatory programme associated with ZIM2 and ZNF549 regulon activity, with notable suppression of the ELK3/NFE2L2 oxidative stress axis operative in the ulcer bed (**Figure 4B**). Quantitative comparison of regulon AUCell scores confirmed that the Ulcer Bed Stroma harboured the highest NFE2L2 activity amongst these five key disease-associated niches (**Figure 4C**), a pattern consistent with the capacity of this niche to persist within a strongly pro-oxidant inflammatory environment. Regulon target-gene network analysis of ELK3 and NFE2L2 further supported direct transcriptional activation of pro-angiogenic effectors (VEGFC, COL1A1) and ECM-remodelling genes, consistent with a link between the upstream TF landscape and the cell-cell communication rewiring and ECM destruction documented in **Figures 2**, **3** (**Figure 4D**). As regulon inference is inherently computational, these master-regulator predictions require confirmation at the protein level (e.g., by multiplex immunofluorescence or immunohistochemistry) before they can be interpreted mechanistically.

**Figure 4:**
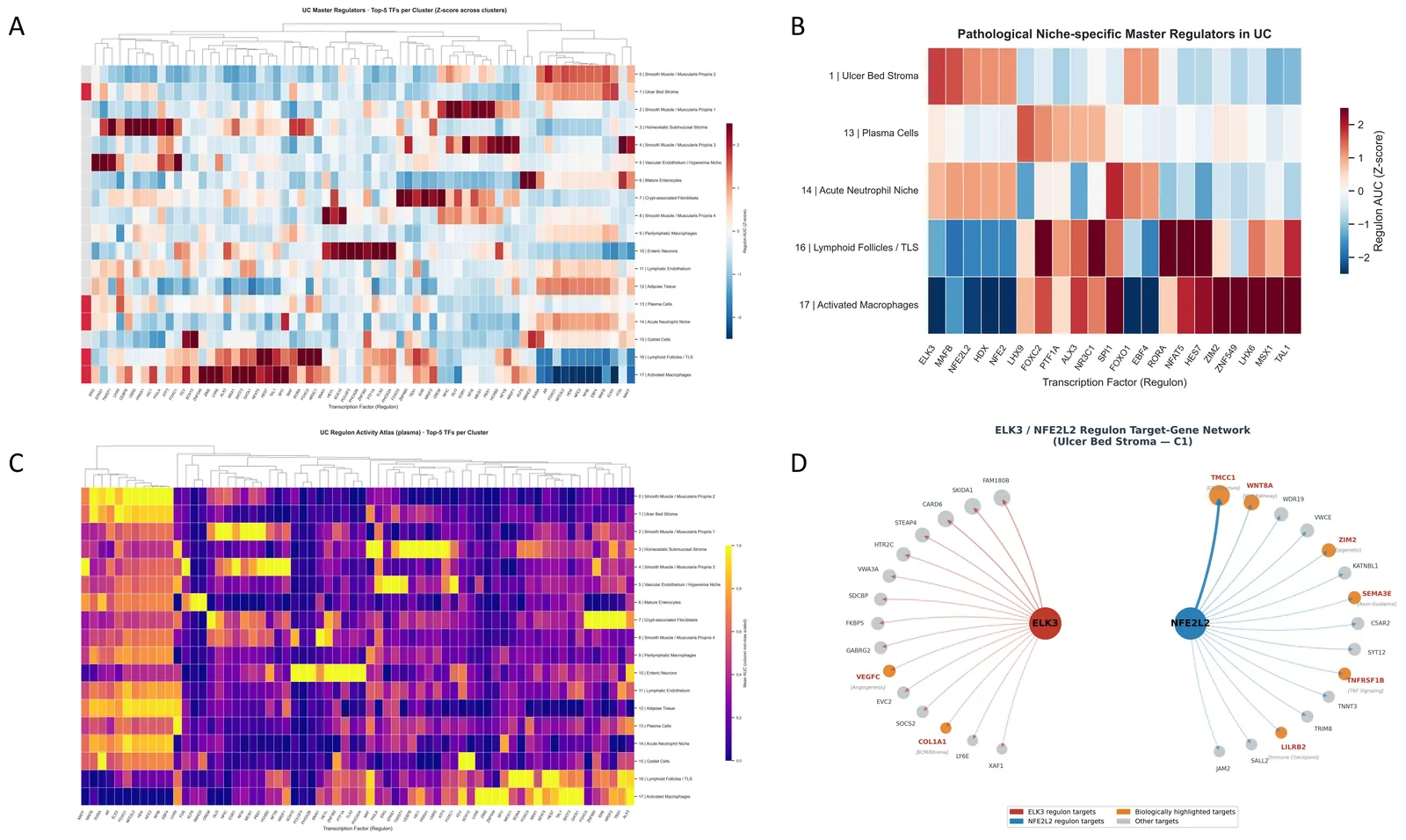
A genome-wide SCENIC regulon atlas identifies niche-specific master transcription factors orchestrating pathological programmes in refractory UC. **(A)** Comprehensive heatmap of top-5 TF regulon activities (AUCell Z-score) for all 18 spatially defined niches, with hierarchical clustering. **(B)** Focused Z-score heatmap for the five key disease-associated niches. **(C)** Raw AUCell values of top-5 TF regulon activities for all 18 niches. **(D)** ELK3/NFE2L2 regulon target-gene network.

### 3.6 Niche signatures associate with non-response across seven cohorts

As a translational extension of the ulcer-bed-centred core discovery, we next assessed whether the niche signatures defined in full-thickness tissue can be detected in bulk mucosal biopsies and whether they associate with treatment outcome.

To assess the translational relevance of our spatially resolved transcriptomic findings, we generated single-sample GSEA (ssGSEA) gene expression signatures for each of the 18 spatially defined niches and systematically evaluated their association with treatment outcomes across seven independent bulk RNA-seq clinical cohorts: six biologic response cohorts encompassing UC patients treated with Infliximab (GSE16879, GSE12251, GSE23597, GSE73661), Golimumab (GSE92415), or Vedolizumab (GSE73661), and one prospective remission cohort from the multicentre PROTECT study (GSE109142; pre-treatment mucosal biopsies from children with newly diagnosed UC, n = 206; endpoint, corticosteroid-free remission at Week 4; **Figures 5A–L**). These signatures are composed of niche-specific marker genes (**Supplementary Table S3**): for example, the Acute Neutrophil Niche signature is dominated by neutrophil effector genes (S100A9, S100A8, CXCL8, CSF3R), the Mature Enterocyte signature by absorptive enterocyte genes (CEACAM5, SLC26A3, FABP1), and the Goblet Cell signature by mucin-related genes (PIGR, MUC12, FCGBP). We acknowledge that some signatures — notably the Acute Neutrophil Niche — partly reflect the baseline mucosal inflammatory burden and cell composition, consistent with their biology; importantly, however, the epithelial signatures (C6, C15) were enriched in responders, i.e., in the direction opposite to inflammatory burden, indicating that the observed associations are not explained by a single inflammation axis alone. A consistent pattern emerged across multiple drug classes and patient populations (**Figures 5A, B**). The Acute Neutrophil Niche (C14) signature was significantly enriched in biologic non-responders in all six response cohorts (p < 0.001 in GSE16879, GSE12251, GSE23597, GSE73661_IFX, GSE92415, and GSE73661_VEDO), supporting it as the most consistently reproduced non-response-associated signature across cohorts and drug classes. The Ulcer Bed Stroma (C1) signature was similarly enriched in non-responders across multiple cohorts and drug classes (GSE16879: p < 0.001; GSE73661_IFX: p < 0.05; GSE73661_VEDO: p < 0.001), indicating that a high ulcerogenic stroma transcriptional burden at baseline is associated with treatment failure for both anti-TNF and anti-integrin agents. The Homeostatic Submucosal Stroma (C3) and Vascular Endothelium/Hyperemia Niche (C5) signatures also demonstrated significant non-response associations across multiple cohorts, highlighting the multi-compartmental nature of treatment resistance in severe UC. Conversely, the Goblet Cell (C15) and Mature Enterocyte (C6) signatures were preferentially enriched in responders (**Figure 5B**), indicating that preserved or recovering epithelial barrier integrity at baseline is a key correlate of therapeutic response (**Figures 5C–F**). In the independent PROTECT paediatric cohort, niche-resolved analysis of pre-treatment biopsies revealed broadly concordant associations (**Figures 5G, H**). The Ulcer Bed Stroma (C1, p < 0.05; **Figure 5I**) and Acute Neutrophil Niche (C14, p < 0.001; **Figure 5I**) signatures were significantly enriched in non-remission patients, with the Acute Neutrophil Niche again demonstrating the strongest discriminating power between remitters and non-remitters (ΔNES = non-remission minus remission) and establishing early mucosal neutrophil transcriptional enrichment as the signature most consistently associated with treatment failure across all cohorts examined. The Vascular Endothelium/Hyperemia Niche (C5, p < 0.05) was also enriched in non-remission patients. In the remission-associated direction, the Mature Enterocyte (C6, p < 0.01; **Figure 5I**) and Goblet Cell (C15, p < 0.05; **Figure 5I**) signatures were significantly enriched in patients achieving remission, whilst Activated Macrophages (C17, p < 0.01) demonstrated a notable remission-associated enrichment, potentially reflecting macrophage repolarisation towards a homeostatic phenotype as a hallmark of successful mucosal healing. Collectively, these cross-cohort, cross-drug-class analyses corroborate that our spatially derived 18-niche transcriptomic framework harbours translational potential as a multi-dimensional molecular framework for studying UC treatment outcome, with the Acute Neutrophil Niche and Ulcer Bed Stroma signatures emerging as particularly promising candidates for prospective evaluation.

**Figure 5:**
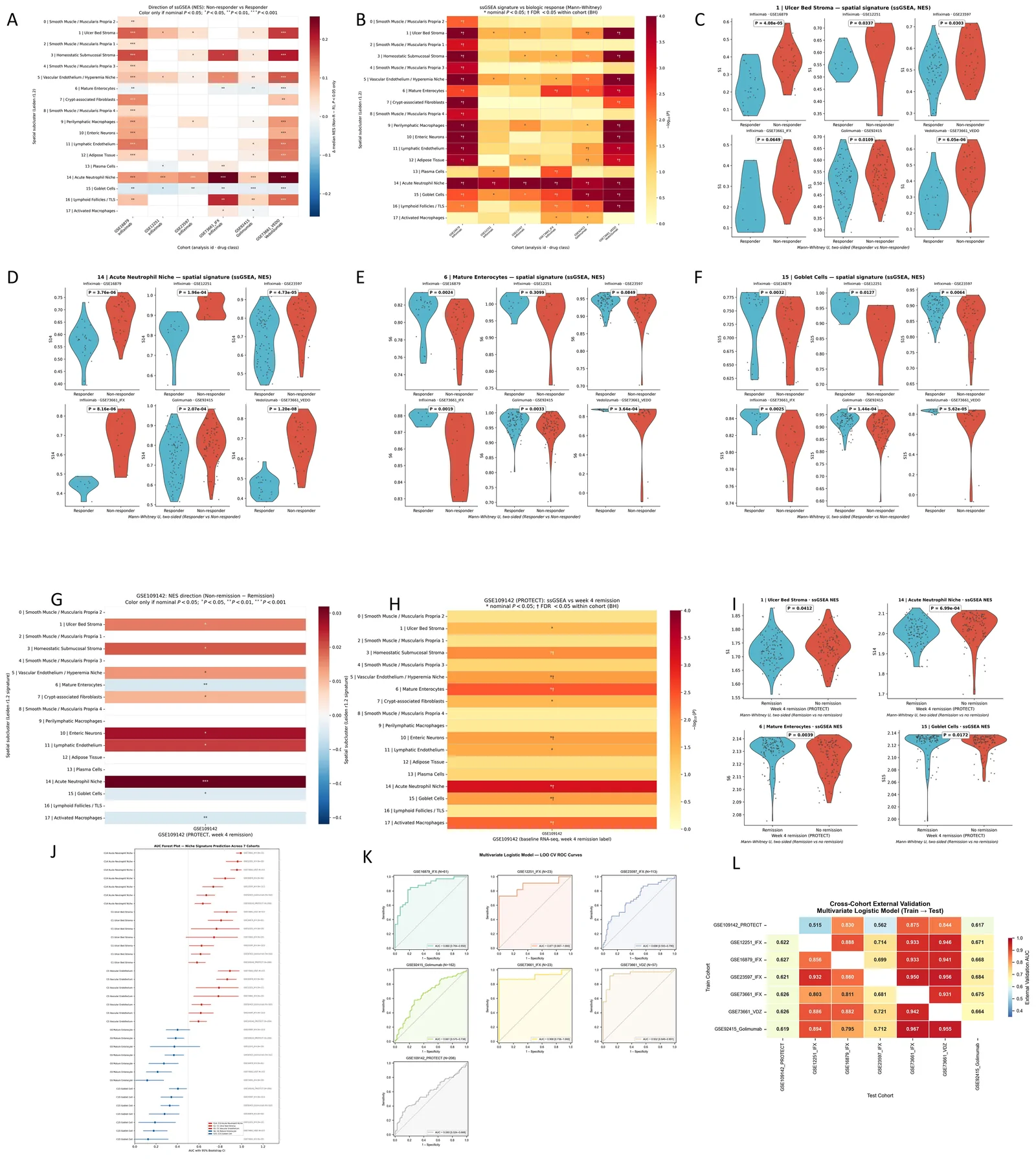
Spatially derived niche transcriptomic signatures associate with biologic non-response and clinical remission across seven independent UC cohorts. **(A)** NES direction heatmap (ΔNES = Non-responder − Responder) for all 18 niche signatures across six biologic therapy cohorts (Infliximab, Golimumab, Vedolizumab). Cells coloured at nominal p < 0.05. **(B)** Significance heatmap (−log10 p). Representative violin plots of ssGSEA enrichment scores for **(C)** Ulcer Bed Stroma (C1), **(D)** Acute Neutrophil Niche (C14), **(E)** Mature Enterocytes (C6), and **(F)** Goblet Cells (C15) in selected biologic cohorts. **(G)** NES direction bar chart for the PROTECT paediatric cohort (GSE109142; pre-treatment biopsies, Week-4 corticosteroid-free remission endpoint). **(H)** Significance bar chart for the PROTECT cohort. **(I)** Composite box plots of ssGSEA enrichment scores for the Ulcer Bed Stroma (C1), Acute Neutrophil Niche (C14), Mature Enterocyte (C6) and Goblet Cell (C15) signatures in remission versus non-remission patients of the PROTECT cohort (GSE109142; pre-treatment mucosal biopsy, Week-4 corticosteroid-free remission; two-sided Mann–Whitney U test). **(J)** Forest plot of single-signature ROC AUCs (point estimate ± 95% bootstrap CI) for the five key niche signatures (C14, C1, C5, C6, C15) across all seven cohorts; dashed line, AUC = 0.5. **(K)** ROC curves of the multivariable five-niche logistic model (C14, C1, C5, C6, C15) evaluated by leave-one-out cross-validation in each cohort; AUC and 95% CI are indicated. **(L)** Cross-cohort external-validation heatmap: each cell shows the AUC obtained when the multivariable model trained in the row cohort is applied, without retraining, to the column cohort. * p < 0.05, ** p < 0.01, *** p < 0.001 (nominal); † FDR < 0.05 within cohort (Benjamini-Hochberg).

To quantify the strength of these associations at the individual-patient level, we evaluated each niche signature as a single-variable classifier of biologic outcome by receiver-operating-characteristic (ROC) analysis with 1,000-fold bootstrap 95% confidence intervals (**Figure 5J**). The Acute Neutrophil Niche (C14) signature discriminated non-responders from responders with high accuracy across the six adult biologic cohorts (AUC = 0.99 [GSE73661-IFX], 0.96 [GSE12251], 0.96 [GSE73661-VDZ], 0.85 [GSE16879], 0.74 [GSE23597] and 0.67 [GSE92415]), and remained associated with non-remission in the paediatric PROTECT cohort (AUC = 0.64). The Ulcer Bed Stroma (C1) and Vascular Endothelium/Hyperemia (C5) signatures showed concordant, drug-class-independent discrimination (AUC up to 0.86 and 0.89, respectively), whereas the epithelial Mature Enterocyte (C6) and Goblet Cell (C15) signatures were inversely associated with non-response (AUC < 0.41), consistent with a response-associated, protective role.

We next combined the five most informative niches (C14, C1, C5, C6 and C15) into a multivariable logistic model and assessed it by leave-one-out cross-validation within each cohort (**Figure 5K**). The composite model achieved cross-validated AUCs of 0.66–0.93 across the six adult biologic cohorts (0.86 [GSE16879], 0.87 [GSE12251], 0.70 [GSE23597], 0.66 [GSE92415], 0.91 [GSE73661-IFX] and 0.93 [GSE73661-VDZ]). To test cross-cohort transferability, we trained the model in each cohort and applied it, without retraining, to every other independent cohort (**Figure 5L**). Amongst the six adult cohorts, cross-cohort AUCs ranged from 0.68 to 0.97 — including robust anti-TNF-to-anti-integrin transfer (e.g. 0.93–0.96) — demonstrating that the niche-based model retains discriminative performance across datasets, platforms and drug classes. Transfer to and from the paediatric PROTECT remission cohort was weaker (AUC ≈ 0.51–0.62), consistent with its distinct population and remission-based endpoint.

We recognise that the cross-cohort performance of the model varied substantially (AUC 0.68–0.97) and was close to chance in the paediatric PROTECT cohort (AUC ≈ 0.51–0.62), indicating that the model currently exhibits population specificity. Three factors likely contribute to this limitation. First, the model was derived from full-thickness tissue of adult patients with end-stage, medically refractory disease; transferability to less severely affected populations is therefore not guaranteed. Second, several component signatures — notably the Acute Neutrophil Niche (C14) — partly reflect the degree of baseline mucosal inflammation, so their discriminative performance is expected to depend on the distribution of disease severity in the population studied. Third, the PROTECT cohort differs from the adult cohorts in both endpoint (corticosteroid-free remission at Week 4 rather than response to a specific biologic agent) and population (largely treatment-naive, newly diagnosed children); although the signatures were enriched or depleted in the expected directions in this cohort (**Figures 5G–I**), their discriminative performance did not transfer. On this basis, we limit the applicability of the present model to adult patients with moderate-to-severe UC under evaluation for biologic therapy; generalisation to the broader UC population — including paediatric, early-stage or treatment-naive patients — is not supported by the current data and requires prospective, population-specific validation.

### 3.7 Cross-species protein-level validation of TNFAIP2+ TLS B cells in DSS colitis

To orthogonally validate the spatial transcriptomic predictions at the protein level, we performed dual-colour immunofluorescence and flow cytometry in a DSS-induced colitis mouse model (3% DSS, 7 days; DSS group n = 5, control n = 5; euthanised on day 10; Animal Ethics No. XMULAC20220195; **Figures 6A, B**). CD19 was selected to identify mature B cells, because in the spatial transcriptomics data the Lymphoid Follicles/TLS niche (C16) is defined by high CD19 and MS4A1 expression alongside CXCL13 and CCL19 (CD19 logFC = +5.64, padj = 1.07×10^-^¹²²), distinguishing it from terminally differentiated, CD19-low plasma cells (C13). CD19+/TNFAIP2+ cells were detected within the inflamed intestinal wall of DSS-colitis mice but were rare in normal controls (**Figure 6A**). Flow cytometric analysis of colon single-cell suspensions confirmed that the proportion of CD19+/TNFAIP2+ cells was significantly elevated in the DSS-colitis group compared with normal controls (p = 0.0079, two-sided Mann–Whitney U test; **Figure 6B**). As an additional computational validation, Tangram-based spatial mapping of deconvolved B_Plasma cell proportions confirmed enrichment of B-lineage cells within the submucosal compartment of UC tissue sections, spatially coincident with the TLS aggregates (**Figure 6C**), providing an independent, transcriptome-wide corroboration of the B-cell spatial patterning predicted by the atlas. Collectively, these cross-species data extend the spatial transcriptomic prediction of TNFAIP2+ TLS-resident B cell expansion; *in situ* protein-level documentation of this population in human tissue remains to be established (see Limitations).

**Figure 6:**
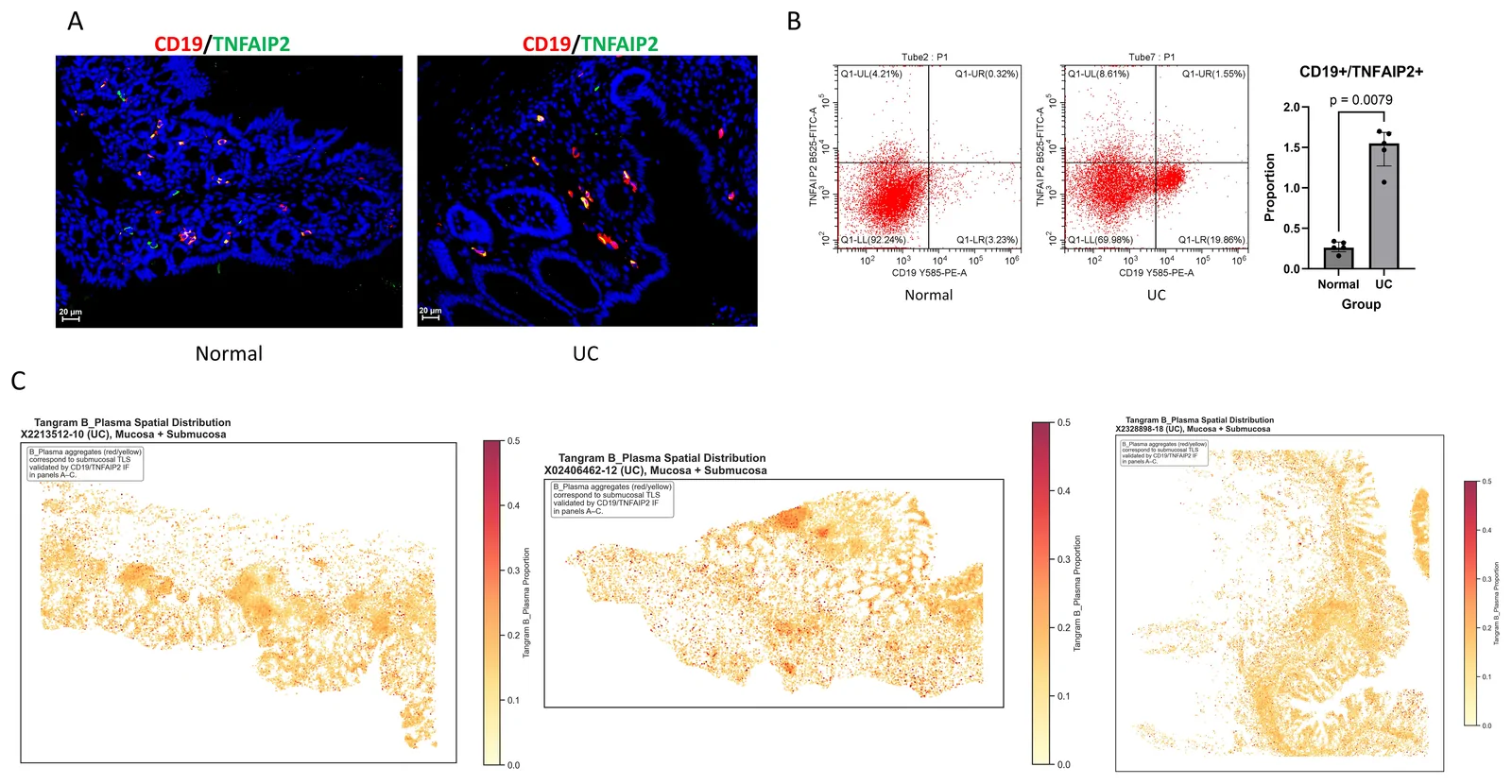
Cross-species protein-level and computational corroboration of TNFAIP2-expressing TLS B cells identified by spatial transcriptomics. **(A)** Dual-colour immunofluorescence of CD19 (red) and TNFAIP2 (green) in DSS-induced colitis C57BL/6J mice versus normal controls; scale bar, 20 µm. **(B)** Flow cytometric analysis of colon single-cell suspensions from DSS-colitis (n = 5) and control (n = 5) mice; the proportion of CD19+/TNFAIP2+ cells was significantly elevated in DSS-colitis mice (p = 0.0079, two-sided Mann–Whitney U test). **(C)** Tangram-deconvolved B_Plasma cell proportions mapped onto representative UC tissue sections (mucosa and submucosa); B_Plasma aggregates localise predominantly to the submucosa, coincident with lymphocytic aggregates in UC. Colour scale: Tangram B_Plasma proportion (0–0.5).

## 4 Discussion

In this study, we generated a high-definition spatial transcriptomics atlas of full-thickness colonic resection specimens from medically refractory UC, resolving 615,274 spatial bins into 18 functionally distinct niches across five hierarchical tissue modules. By integrating zone-stratified differential expression, spatial cell-cell communication analysis, SCENIC-based regulon inference (9), and a Squidpy-powered (10) spatial framework, our study maps the spatial architecture of pathological remodelling across all layers of the severely inflamed colon wall. Niche-derived transcriptional signatures showed reproducible associations with biologic non-response and clinical remission across seven independent clinical cohorts spanning anti-TNF and anti-integrin drug classes, suggesting a translational link between the spatial microenvironment and clinical outcome.

A central finding of this study is the identification and spatial characterisation of the Ulcer Bed Stroma niche (C1) as the dominant pathological entity in the refractory UC mucosa, expanding from 0.91% in normal colon to 18.36% in UC (p = 0.030) and exhibiting massive upregulation of tissue-destructive MMPs (MMP1, MMP3, MMP7). This observation is consistent with prior biochemical evidence that MMP activity is a principal driver of mucosal degradation and barrier breakdown in IBD (19). The Acute Neutrophil Niche (C14), which spatially co-localises with the ulcer bed, is positioned to amplify this destructive cascade: neutrophil elastase-mediated degradation of type IV collagen in the intestinal basement membrane has been established as an early determinant of mucosal destruction in UC (20), and our data show that the Acute Neutrophil Niche (C14) is markedly expanded and spatially co-localised with the ulcer bed, in association with CXCL12–CXCR4 signalling, in refractory disease. This stromal and neutrophilic expansion is accompanied by a depletion of the epithelial barrier-forming populations — Mature Enterocytes (C6, −10.55%, p = 0.004) and Goblet Cells (C15, −13.96%, p = 0.004) — recapitulating in spatially resolved detail the epithelial identity collapse that characterises refractory UC. The depletion of goblet cells and their protective mucus barrier function is a hallmark of UC pathogenesis. A recent study demonstrated that IFN-γ signalling drives apoptotic extrusion of BMP2+ surface colonocytes and triggers compensatory stromal niche remodelling mediated by HGF, representing a reparative response to acute epithelial injury (21). Our findings suggest that in refractory UC, this reparative programme is overwhelmed and replaced by a pathological ulcer bed stroma associated with ongoing tissue destruction rather than regeneration — a transition from acute repair to chronic, irreversible remodelling.

The Vascular Endothelium/Hyperemia Niche (C5) was significantly expanded in the UC mucosa (p = 0.004), and our ligand-receptor analysis identified the VEGFA–CDH5 axis as a key signalling pathway emanating from the Ulcer Bed Stroma (C1) towards this vascular niche. This finding provides spatially resolved correlative support for the established observation that VEGFA drives angiogenesis and promotes a pro-inflammatory vascular phenotype in IBD, including enhanced neutrophil adhesion to intestinal endothelium (22). The spatial co-localisation of the Ulcer Bed Stroma, Acute Neutrophil Niche, and Vascular Endothelium/Hyperemia Niche at the mucosal frontline — as validated by neighbourhood enrichment analysis — outlines a tri-niche ulcerogenic axis not previously resolved in full-thickness tissue in which angiogenic, fibrotic, and inflammatory signals are spatially integrated, potentially perpetuating ulcer formation and impairing epithelial regeneration.

Our SCENIC regulon analysis identified NFE2L2 as a master transcription factor specifically hyperactivated in the Ulcer Bed Stroma (C1), with cross-niche AUCell quantification showing this niche held the highest NFE2L2 regulon activity amongst the five key disease-associated niches profiled. This finding is consistent with prior evidence that NRF2 (encoded by NFE2L2) is activated in colonic epithelial cells exposed to reactive oxygen species from IBD inflammatory cells, conferring apoptosis resistance through proteasome upregulation (23). Alongside NFE2L2, our analysis identified ELK3 as a co-hyperactivated master regulator in the Ulcer Bed Stroma, with its top-ranked target genes including VEGFC and COL1A1. ELK3/Net has been established as a pivotal regulator of the angiogenic switch that directly drives VEGF promoter activity and promotes angiogenesis in response to hypoxic and Ras-mediated signals (24). Our data are thus consistent with a link between the hypoxic ulcer bed microenvironment and pathological angiogenesis, potentially mediated by ELK3-associated transcriptional regulation — providing a plausible molecular link between SCENIC regulon findings and the VEGFA–CDH5 communication axis identified in **Figure 3**. Together, NFE2L2 and ELK3 hyperactivation constitutes a dual transcriptional survival-and-destruction programme consistent with the capacity of the Ulcer Bed Stroma to persist and expand in the extreme oxidative, hypoxic microenvironment of the UC ulcer, rather than undergoing apoptotic clearance — partially accounting for the resistance of refractory UC lesions to therapeutic intervention.

Beyond the mucosal frontline, the submucosa revealed an integral component of the same atlas: a humoral immune programme characterised by massive immunoglobulin gene upregulation (IGHA1, IGKC, IGHG1, IGHG3, MZB1) and the spatial emergence of structured Tertiary Lymphoid Structures (TLS, C16) within the deep submucosal stroma. The marked upregulation of MZB1 — an ER-resident chaperone that facilitates immunoglobulin assembly and IgA secretion and whose deficiency causes severe intestinal colitis in mice (25) — underscores the centrality of the antibody secretory machinery in the submucosal pathology of refractory UC. The accompanying Unfolded Protein Response (UPR) enrichment observed in our GSEA is mechanistically consistent with the known requirement for XBP1-mediated UPR activation to support the extraordinary immunoglobulin secretory capacity of terminally differentiated plasma cells (26). TLS formation is an established pathological feature of chronic intestinal inflammation, and the debate as to whether intestinal TLS are protective or perpetuate disease remains unresolved. Our data provide spatially resolved insights into this question: our ligand-receptor analysis shows that the TLS (C16)–Plasma Cell (C13) axis is dominated by survival-related signals (MIF–CD74 (anti-apoptotic (27)) and LTB–CD40 (B cell differentiation)), consistent with high-volume immunoglobulin production despite global communication collapse. This suggests that in refractory UC, submucosal TLS may function primarily as survival hubs for antibody-secreting plasma cells, potentially perpetuating the humoral immune storm rather than resolving it — a finding with important implications for understanding why patients with TLS-positive, immunoglobulin-high disease may be refractory to conventional therapies. Our Tangram-based deconvolution analysis provides an orthogonal, cell-type-resolved lens that complements the niche-level analyses. The concordance between deconvolved cell-type proportions and transcriptionally defined niche identities supports both approaches, whilst the independent spatial confirmation of B-lineage enrichment in submucosal TLS regions reinforces the IF and flow cytometry findings. Furthermore, full-gene LIANA analysis confirmed that the ECM-dominated intercellular communication landscape is not attributable to HVG selection bias but reflects the genuine sparsity of cytokine mRNAs in Visium HD spatial data, lending methodological confidence to the niche-level L-R findings.

Perhaps the most translational observation of this study is that spatially derived niche gene signatures consistently associate with biologic treatment outcomes across seven independent clinical cohorts. The Acute Neutrophil Niche (C14) signature was significantly enriched in non-responders in all six biologic response cohorts (p < 0.001 across anti-TNF and anti-integrin (vedolizumab (4)) cohorts) and was the most strongly enriched signature in non-remission patients of the PROTECT paediatric cohort (p < 0.001), identifying mucosal neutrophil transcriptional burden as the signature most consistently associated with treatment failure across drug classes; the multivariable model, however, did not transfer to this paediatric cohort (Section 3.6). Similarly, the Ulcer Bed Stroma (C1) signature was associated with non-response across drug classes. Conversely, preserved or recovering Mature Enterocyte (C6) and Goblet Cell (C15) signatures were consistently enriched in responders, underscoring epithelial barrier integrity as a fundamental determinant of therapeutic response. These findings complement and spatially contextualise prior scRNA-seq evidence associating inflammatory fibroblast subtypes with anti-TNF non-response (6), and prior spatial imaging data identifying fibroblast-monocyte modules as predictors of vedolizumab non-response (14), by providing a spatially and mechanistically coherent multi-niche framework applicable across multiple drug classes.

Several limitations of the current study warrant discussion. First, the discovery cohort is small, comprising three UC patients and three normal controls. Full-thickness colonic resection specimens from patients with end-stage, medically refractory UC are rare, and obtaining additional specimens was not feasible within the study period. We have therefore deliberately framed this study as a hypothesis-generating spatial atlas: findings derived from the n = 3 discovery cohort are descriptive, whilst the statistical power for outcome associations is provided by the seven external clinical cohorts, in which the key niche signatures showed reproducible associations with biologic response in the expected directions. We further acknowledge that the control specimens were not typical “healthy” donors: all three controls were normal colon specimens resected for non-IBD indications and confirmed histologically normal, with no history of IBD (Mayo score 0), one of which (a 66-year-old female donor) had a body mass index of approximately 15.0 kg/m², consistent with severe underweight, which we disclose in **Supplementary Table S1**. We have accordingly avoided the term “healthy controls” and refer to these specimens as normal controls throughout the manuscript; the term “normal” used here refers to the tissue phenotype (histologically non-inflamed colon), not to the donors’ overall health status. Prospective studies in larger, well-characterised cohorts will be required to confirm the generalisability of the atlas findings. Second, all UC specimens represent medically refractory disease requiring total colectomy, which may not fully reflect the transcriptomic landscape of earlier or treatment-naive UC. Third, the cross-sectional design of spatial transcriptomics precludes direct assessment of the dynamic temporal progression of pathological niche formation. Fourth, cross-species protein-level evidence for TNFAIP2-expressing TLS B cells (C16) is provided by immunofluorescence and flow cytometry in a DSS colitis model (p = 0.0079; **Figures 6A, B**), whereas *in situ* protein-level confirmation of this population in human tissue remains to be established. Moreover, the NFE2L2/ELK3 regulon activities were not validated at the protein level; as SCENIC inference is a computational prediction, direct *in situ* confirmation of NFE2L2/ELK3 protein expression in the ulcer bed stroma and of the predicted TF-target relationships by multiplex immunofluorescence or immunohistochemistry is required before these findings can be interpreted mechanistically. Fifth, the generalizability of the model is limited. Cross-cohort performance varied widely (AUC 0.68–0.97), and transfer to the paediatric PROTECT cohort approached chance (AUC ≈ 0.51–0.62), indicating that the model exhibits population specificity and cannot yet be considered applicable to the broader UC population. As detailed in Section 3.6, this likely reflects the derivation of the signatures from adult end-stage disease, the partial dependence of several signatures on baseline inflammatory burden, and the distinct endpoint and population of PROTECT. The present model should therefore be regarded as applicable only to adult patients with moderate-to-severe UC under evaluation for biologic therapy; its value in other patient groups, and beyond established markers of disease severity, warrants prospective evaluation. Future prospective longitudinal spatial profiling of UC across multiple disease stages and treatment time points will be important to translate these findings into dynamic biomarker frameworks.

Sixth, ligand-receptor interaction analysis was performed at the level of 16-µm Visium HD bins. At this resolution, the boundaries of adjacent cells cannot be resolved, and co-expression of a ligand and its receptor within the same bin does not formally distinguish intercellular signalling from autocrine signalling or from interactions amongst cells captured within the same bin. Although the reported interactions were filtered by permutation-based significance testing and key spatial relationships were independently supported by neighbourhood enrichment analysis, the ligand-receptor results should be regarded as hypothesis-generating; confirmation at single-cell or subcellular resolution (e.g., subcellular spatial transcriptomics or multiplex imaging) or by functional experiments will be required.

In conclusion, this study provides a comprehensive high-definition spatial transcriptomic atlas of the refractory UC colon that reveals the multi-layer, spatially integrated pathological programmes associated with mucosal barrier collapse, submucosal immune remodelling, and tissue communication rewiring in medically refractory UC. By anchoring transcriptional findings to their precise spatial context within the full colon wall thickness, we describe the spatial architecture of refractory UC at a resolution not previously available. The reproducible association of spatially derived niche signatures with biologic therapy outcomes across multiple drug classes and patient populations positions this framework as a promising resource for the development of spatially grounded candidate biomarkers and, ultimately, for the rational design of niche-targeted therapeutic strategies in treatment-refractory UC.

At its core, this atlas demonstrates that the medically refractory UC colon is organised around a single pathological unit — the ulcer-bed-centred tri-niche axis at the mucosal frontline — whose transcriptional signatures carry reproducible clinical associations.

## Data Availability

The datasets presented in this study can be found in online repositories. The names of the repository/repositories and accession number(s) can be found below:https://www.ncbi.nlm.nih.gov/, PRJNA1528864.

## Supplementary material

Supplementary Table 1 Sample-level clinical and demographic characteristics of the six Visium HD specimens used in this study (gender, age, height, weight, BMI, Mayo score and endoscopic Mayo subscore), together with per-sample raw bin counts after quality control.

Supplementary Table 2 Per-niche spatial spot composition by disease group. For each of the 18 spatially defined niches (Clusters 0–17; **Figure 1A**), the number and proportion of Visium HD bins originating from UC (n = 3) and normal colon (n = 3) specimens are listed across all four histological zones. All 18 niches were detected in both disease groups; the three Pathological Core niches (C1, C14, C16) were markedly expanded in UC, whereas the epithelial niches (C6, C15) were enriched in normal colon.

Supplementary Table 3 Niche signature gene lists used for single-sample GSEA in the external cohorts. For each of the 18 spatially defined niches, the marker genes (up to 30) derived from the niche-level differential expression analysis are listed. The Acute Neutrophil Niche (C14) signature comprises 21 genes; all other signatures comprise 30 genes. These gene lists were used to compute ssGSEA enrichment scores in the seven external cohorts.

Supplementary Figure 1 A. UMAP embedding of all spatial spots coloured by histological zone (Mucosa, Submucosa, Muscularis Propria, Serosa) — formerly **Figure 1D**. Zones were assigned via pathologist-annotated H&E registration (QuPath v0.5). B. Heatmap of mean Tangram-deconvolved cell-type proportions per niche (18 niches x 11 cell types; Z-score across niches) — formerly **Figure 1H**. Red indicates enrichment of a cell type relative to the niche average; blue indicates depletion.
